# PEBAX-1657/Ag nanoparticles/7,7,8,8-tetracyanoquinodimethane complex for highly permeable composite membranes with long-term stability

**DOI:** 10.1038/s41598-019-40185-6

**Published:** 2019-03-12

**Authors:** Minsu Kim, Sang Wook Kang

**Affiliations:** 1 Department of Chemistry, Sangmyung University, Seoul, 03016 Republic of Korea; 2 Department of Chemistry and Energy Engineering, Sangmyung University, Seoul, 03016 Republic of Korea

## Abstract

Poly (ether-block-amide) resin-1657 (PEBAX-1657)/silver nanoparticle (AgNPs)/7,7,8,8-tetracyanoquinodimethane (TCNQ) composite membranes were prepared for olefin/paraffin separation. The long-term performance of composite membranes can be improved by preventing the reduction of silver ions by adding Al(NO_3_)_3_ and PEBAX-1657, which has 40% amide groups and 60% ether groups, to the polymer matrix for high permeance. In this study, silver ions were reduced to form nanoparticles to which 7,7,8,8-tetracyanoquinodimethane (TCNQ, electron acceptor) was added to produce long-term olefin carriers. The surface of the AgNPs were modified using electron TCNQ to induce positive charges. The polarized surface of the AgNPs in the PEBAX-1657 permeable polymer matrix interacted with olefins. The membrane was expected to show exceptional separation performance. The results showed that the PEBAX-1657/AgNPs/TCNQ composite membrane exhibited a selectivity of 12.7 and a mixed-gas permeance of 10.2 GPU for durations longer than 76 h. The surface-activated AgNPs were characterized using infrared spectroscopy and X-ray photoelectron spectroscopy.

## Introduction

With an increase in the demand for synthetic resins, fibers, and rubbers, the petrochemical industry that produces the raw materials for these products is becoming increasingly large-scale^1^. The separation of olefin/paraffin mixtures is one of the most important processes in the petrochemical industry. Currently, this separation is mainly carried out by cryogenic distillation processes that require significant capital investment and high operating costs because of chemical and physical similarities of olefin and paraffin^2–4^. Therefore, new technologies such as ZIF-4, ZIF-8 and MOF are being studied for olefin/paraffin separation to replace inefficient processes^5–15^.

Over the past few years, polymer/silver ion electrolyte membranes have been gained immense attention for their separation applications owing to their selectivity and permeance^16–18^. When silver salts such as AgBF_4_ or AgClO_4_ are dissolved in polar polymers such as polyvinylpyrrolidone (PVP), poly(ethylene oxide) (PEO), or polymethacrylates (PMA), free silver ions are produced through the formation of solid polymer electrolytes^19–24^. However, silver ion-based solid polymer electrolyte membranes are not as efficient as the distillation separation method for separating olefin/paraffin mixtures. In particular, polar polymers such as poly(2-ethyl-2-oxazoline) (POZ), PEO, and PVP act as the reducing agents for silver ions, which are easily reduced to nanoparticles. This deteriorates the separation performance of solid polymer electrolyte membranes with silver ions. To overcome this limitation of silver ion-based polymer membranes, a transport method using positively charged silver nanoparticles capable of reacting reversibly with olefins has been developed recently^25–28^. The carriers form a reversible complex with specific components and facilitate their transport^29^.

This method not only offers improved selectivity and permeance but also results in a stable performance because it utilizes silver ions that have already been reduced. Hence, this concept of the transfer of reversible complex components by silver nanoparticles has become very popular for the development of membranes with high permeance. In order to overcome the limitations of silver ion-based polymer membranes, we developed a composite membrane utilizing silver nanoparticles in this study.

In a previous study, we reported that AgNPs can be positively polarized by *p*-benzoquinone (*p*-BQ), which is an electron acceptor and is used as an olefin carrier. The PVP/AgNPs/*p*-BQ composite membrane exhibited a propylene/propane selectivity of 1 and a total gas permeance of 0.8 GPU (1 GPU = 1  × 10^−6^ cm^3^ (STP)/(cm^2^ s cm Hg))^30^.

On the other hand, we recently reported the excellent separation performance of poly(vinylpyrrolidone) (PVP)/AgNPs nanocomposite membranes utilizing the interface of the dipole on the surface of AgNPs with 7,7,8,8-tetracyanoquinodimethane (TCNQ), which is a strong electron acceptor. The membranes exhibited unprecedented separation performance: a selectivity of 50 and a permeance of 3.5 GPU^31,32^. This demonstrated that positively charged AgNPs can perform well as an olefin carrier for the separation of olefin and paraffin. However, the membranes that can be used for industrial processes require high permeance. Hence, polymers offering high permeance should be utilized to develop separation membranes. In this study, PEBAX-1657 was utilized to increase the permeance. Poly(ether-block-amide) resin is known as the trademark PEBAX, which is a thermoplastic elastomer consisting of linear chains. PEBAX consists of two segments: hard and soft segments^33^. The hard polyamide segment offers mechanical strength to the membranes, while the flexible polyether segment offers high permeance because of high chain mobility of the ether linkage. Hence, the PEBAX-1657/AgNPs/TCNQ composite membrane showed enhanced permeance as well as long-term stability.

## Results and Discussion

### Scanning electron microscopy (SEM)

**Figure 1:**
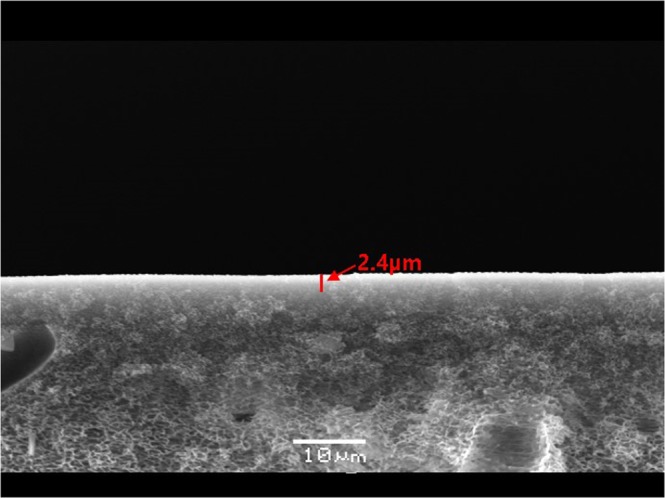
SEM image of the PEBAX-1657/AgNPs/TCNQ membrane.

The selective layer thickness and surface state of the PEBAX-1657/AgNPs/TCNQ membrane were determined from its SEM images of Fig. 1. The SEM analysis revealed that the solution was uniformly coated on the film. The thickness of the selective layer was approximately 2.4 µm. The structure of the polysulfone support was sponge-like.

### Separation Performance

To investigate the effect of flexible PEBAX-1657 on the permeance of the membrane, a long-term mixed gas permeation experiment was conducted. Table 1 gives the selectivity and permeance of the 1/6.67/0.0167 (wt ratio) PEBAX-1657/AgNPs/TCNQ membrane for propylene/propane mixed gas^30^. The PEBAX-1657/AgNPs/TCNQ membrane showed a propylene/propane selectivity of approximately 12.7 and an improved gas permeance of 10.2 GPU. This increase in the permeance was caused by the stabilization of AgNPs by the polyamide group of the PEBAX-1657 matrix and the high chain mobility of the ether linkages in the polyether group of PEBAX-1657. For this reason, the polarized AgNPs were capable of reversibly interacting with propylene molecules, leading to their accelerated transport.

**Table 1:** Gas separation performance of PVP/AgNPs/TCNQ and PEBAX-1657/AgNPs/TCNQ.

	selectivity	permeance (GPU)
PVP/AgNPs/TCNQ^31^	50	3.5
PEBAX-1657/AgNPs/TCNQ	12.7	10.2

**Figure 2:**
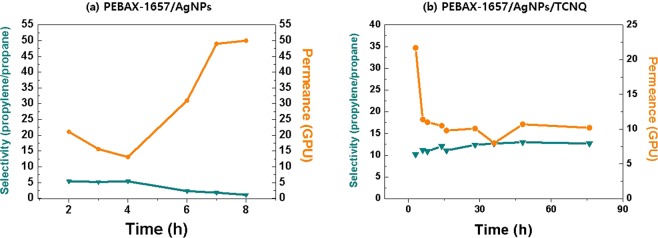
Selectivity and permeance performance of (**a**) PEBAX-1657/AgNPs and (**b**) PEBAX-1657/AgNPs/TCNQ.

Fig. 2 represented the selectivity and permeance performance of PEBAX-1657/AgNPs and PEBAX-1657/AgNPs/TCNQ. In the system of AgNPs without an electron acceptor, AgNPs did not act as an olefin carrier and acted as a barrier, so that initial selectivity was relatively low. The observed initial separation performance was the result of some unreduced ions. However, performance continues to decrease over time by continuously reduced Agion. On the other hand, it was confirmed that the addition of the electron acceptor TCNQ enabled the surface-polarized AgNPs to act effectively as an olefin carrier and maintained the performance for 76 hours.

**Figure 3:**
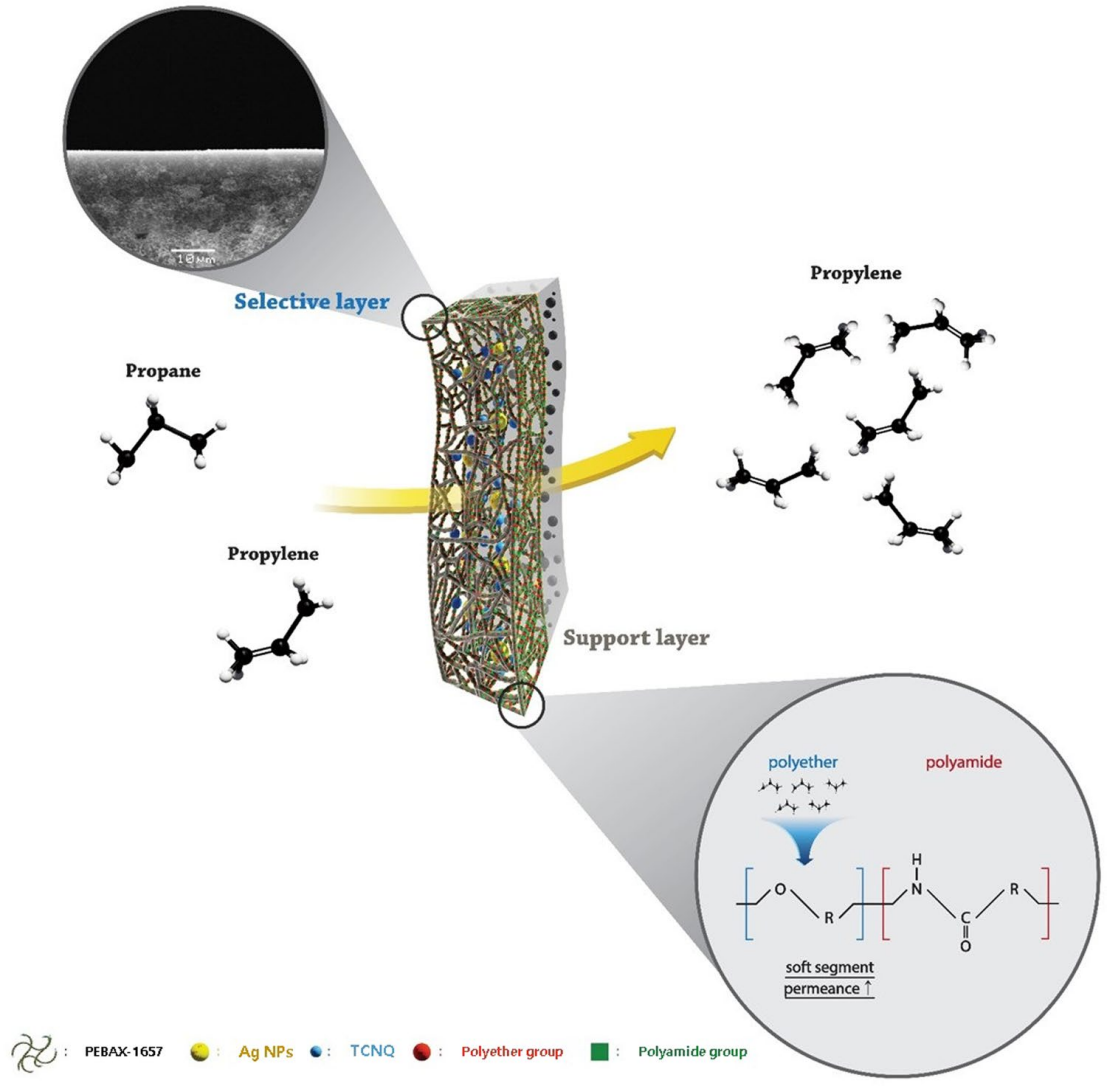
Gas separation of PEBAX-1657/AgNPs/TCNQ nanocomposite membrane.

The gas separation performance of the nanocomposite membranes containing AgNPs activated by TCNQ is shown in Fig. 3. This scheme indicates that the AgNPs polarized by TCNQ were well-dispersed into PEBAX-1657 and cast onto the microporous PSF membrane support. Those polarized AgNPs selectively separates propylene gas as an olefin carrier. In addition, polyether group of PEBAX-1657 is a soft segment that enhances the permeance of gas.

### FT-IR analysis

**Figure 4:**
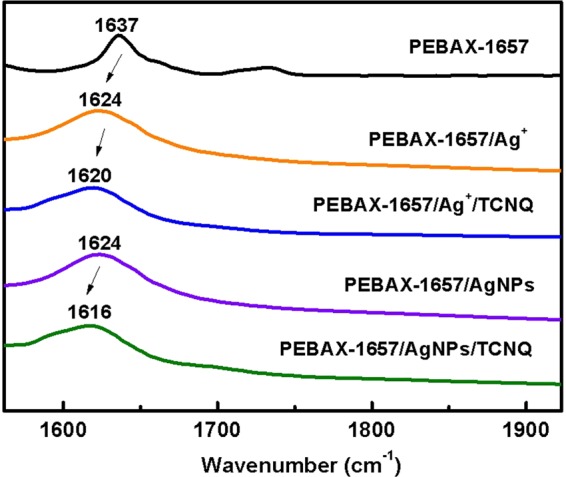
FT-IR spectra of carbonyl group peaks of PEBAX-1657/Ag^+^, PEBAX-1657/Ag^+^/TCNQ, PEBAX-1657/AgNPs, and PEBAX-1657/AgNPs/TCNQ.

The interactions between silver ions/particles and PEBAX-1657 were investigated by FT-IR spectroscopy. The FT-IR spectra of PEBAX-1657/AgBF_4_ and PEBAX-1657/AgBF_4_/TCNQ are shown in Fig. 4. Free C=O (carbonyl bonds in the polyamide group) stretching bands of neat PEBAX-1657 were observed at 1637 cm^−1^. When Ag ions by AgBF_4_ were incorporated into the membrane, the C=O stretching band shifted to 1624 cm^−1^. This shift is attributed to the weakening of the C=O bond because of the electron donation from the C=O bond to Ag ions. similarly, When AgNPs were added to PEBAX-1657 stretching bands of C=O shifted from 1637 to 1624 cm^−1^. This suggests that the interaction of remaining Ag ions not reduced to AgNPs still interacted with carbonyl group. However, in the long-term experiment, the performance did not come out when it was reduced to AgNPs. The reason is that some were still present as ions but were due to the barrier effect by the already reduced particles. On the other hand, introduction of TCNQ into the composite membrane leads to different shift changes. Addition of TCNQ to PEBAX-1657/AgBF_4_ resulted in a shift from 1624 cm^−1^ to 1620 cm^−1^, while, addition of TCNQ to PEBAX/AgNPs resulted in a larger shift from 1624 cm^−1^ to 1616 cm^−1^.

**Figure 5:**
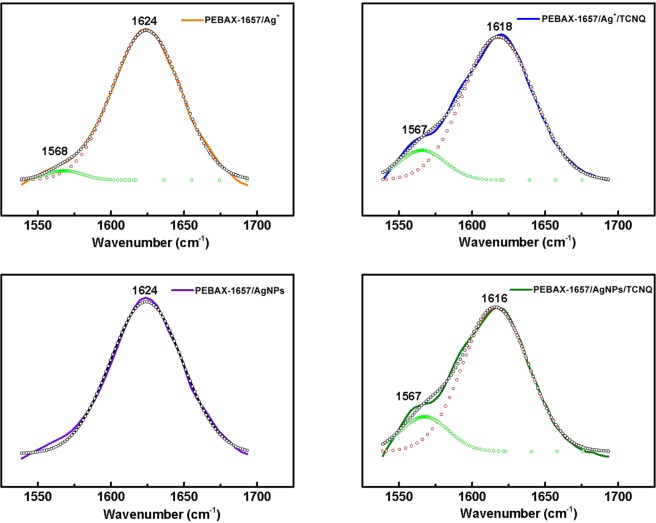
Deconvoluted FT-IR spectra of (**a**) PEBAX-1657/Ag^+^, (**b**) PEBAX-1657/Ag^+^/TCNQ, (**c**) PEBAX-1657/AgNPs, and (**d**) PEBAX-1657/AgNPs/TCNQ.

The FT-IR spectra of the membranes were deconvoluted to elucidate the peak shift of the carbonyl groups in Fig. 5. In the case of silver ions, the intensity of the peak between 1616 and 1624 cm^−1^ decreased from 96.73% to 87.95% after the addition of TCNQ as shown in Table 2. On the other hand, a larger difference (from 100% to 85.30%) was observed in the case of AgNPs as shown in Table 3. This indicates that the silver ions reduced to particles and which polarized by addition of TCNQ resulted stronger interaction with the carbonyl group. In addition, the particles were effectively stabilized by the polyamide group of the hard segment of PEBAX-1657.

**Table 2:** Area percentage of each FT-IR peak for the PEBAX-1657/AgBF_4_ and PEBAX-1657/AgBF_4_/TCNQ complexes.

	1578–1568 cm^−1^ (area %)	1616–1624 cm^−1^ (area %)
PEBAX-1657/Ag^+^	3.27	96.73
PEBAX-1657/Ag^+^/TCNQ	12.05	87.95

**Table 3:** Area percentage of each FT-IR peak for the PEBAX-1657/AgNPs and PEBAX-1657/AgNPs/TCNQ composites.

	1578–1568 cm^−1^ (area%)	1616–1624 cm^−1^ (area %)
PEBAX-1657/AgNPs	0	100
PEBAX-1657/AgNPs/TCNQ	14.70	85.30

### TGA analysis

**Figure 6:**
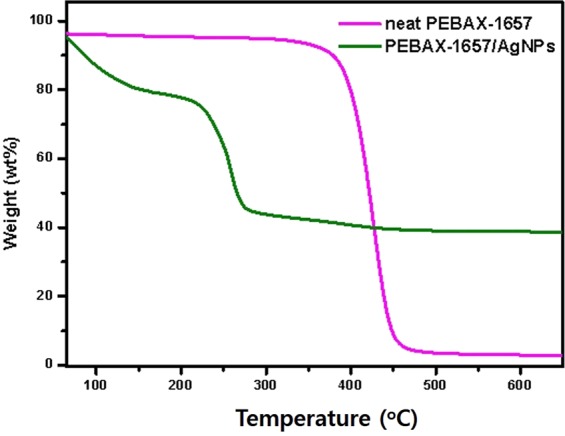
TGA graph of PEBAX-1657 and PEBAX-1657/AgNPs.

The thermal properties of PEBAX-1657 and PEBAX-1657 AgNPs composite membranes were investigated by TGA. As shown in Fig. 6, unlike neat PEBAX-1657, the membrane with AgNPs showed the initial weight loss at 80–120 °C. The second weight loss occurred at 230–250 °C, degrading the PEBAX-1657 membrane. When AgNPs were incorporated into the PEBAX-1657 polymer, the AgNPs penetrated between the PEBAX chains, increasing the free volume of the polymer chain by mutual interactions. This reduced the thermal stability of the PEBAX/AgNPs composite membrane. These results suggest that the PEBAX/AgNPs composite membrane showed an improved permeance because of the high free volume of the polymer chain in it.

### XPS analysis

**Figure 7:**
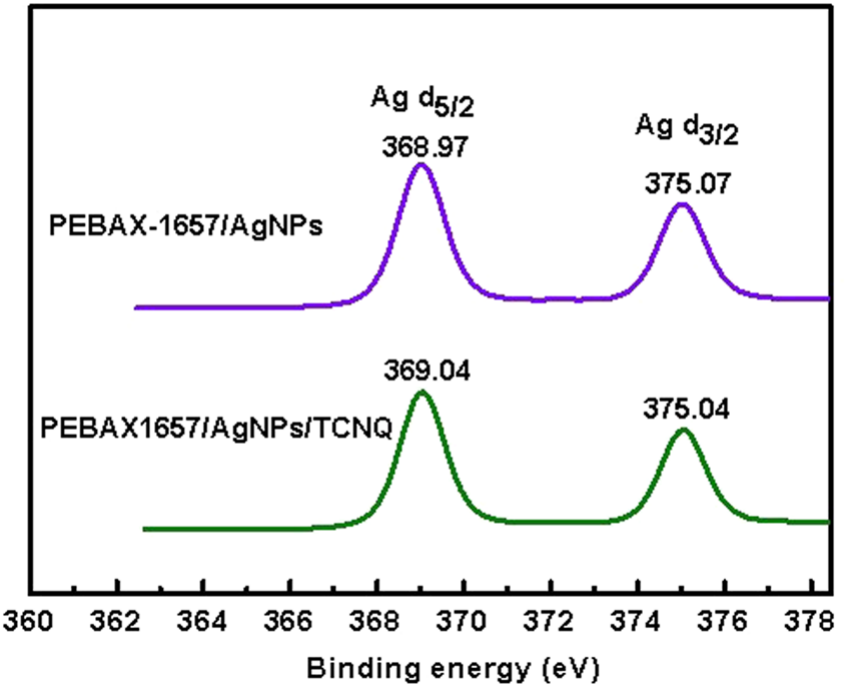
XPS spectra of the PEBAX-1657/AgNPs and PEBAX-1657/AgNPs/TCNQ complexes showing the binding energies of silver particles.

The changes in the chemical environment around AgNPs-TCNQ were analyzed by X-ray photoelectron spectroscopy (XPS). Figure 7(a) shows the Ag 3d_5/2_ regions of the XPS spectra of the PEBAX-1657/AgNPs and PEPAX1657/AgNPs/TCNQ composite membranes. For PEBAX-1657/AgNPs, the Ag 3d_5/2_ peak was observed at 368.97 eV. When TCNQ was added to PEBAX-1657/AgNPs, the binding energy increased from 368.97 to 369.04 eV, indicating that AgNPs were positively polarized by TCNQ. The relatively small decrease in the binding energy can be attributed to the presence of Ag^+^ in TCNQ^− ^^25^.

In conclusion, the PEBAX-1657/AgNPs/TCNQ composite membrane showed a high permeance for olefin/paraffin separation. TCNQ, which is an olefin carrier improved the permeance of the membrane by producing a high positive charge on the surface of the AgNPs. The stabilization of the AgNPs by the polyamide segment of PEBAX-1657 and the high flexibility of polyether, (the soft segment of PEBAX-1657) also contributed to the high permeance of the membrane.

## Methods

### Synthesis of silver nanoparticles

The AgNPs were prepared in a fixed weight ratio of 1/6.67 PEBAX-1657/AgBF_4_. The solution was stirred for 30 min at 75 °C. The color of the solution (transparent) changed to pale yellow.

### Surface activation via TCNQ

TCNQ (Sigma-Aldrich) was added to the chemically synthesized AgNPs dispersed in an ethanol/H_2_O solution with PEBAX-1657. The resulting mixture was stirred at room temperature for 2 days. The color of the solution changed to dark blue. This solution was then coated onto microporous polysulfone membrane supports (Toray Chemical Inc., Korea) using an RK control coater (Model K202, Control Coater RK Print-Coat Instruments Ltd., UK). The resulting membrane was then vacuum dried for 24 h.

### Gas separation experiments

The PEBAX-1657/AgNPs/TCNQ complex was dried in a vacuum oven for one day at room temperature. The flow rates of the mixed gas were controlled using mass flow controllers. The gas permeance values were measured using a bubble flow meter upstream at various pressures (3 bar) and downstream at atmospheric pressure. Their propylene/propane selectivities (50:50 vol% propylene:propane mixture) were measured using gas chromatography (Young Lin 6500 GC system). The gas permeance was expressed in units of GPU, where 1 GPU = 1 × 10^−6^ cm^3^ (STP)/(cm^2^·s·cm Hg).

### Characterization

The selective layer thickness and surface state were determined by scanning electron microscopy (SEM, JEOL JSM-5600LV). The infrared (IR) measurements were performed using a VERTEX 70 Fourier transform (FT)-IR spectrometer. For the IR measurements, the signals between 64 and 200 scans were averaged with a resolution of 4 cm^−1^. The X-ray photoelectron spectroscopy (XPS) analysis was carried out using a K-alpha system (Thermo Scientific Inc., UK). The spot size was 400 µm. The 285.0 eV carbon (C 1 s) line was used as the reference. The weight loss of the composite membrane under the flow of N_2_ was determined by carrying out its thermogravimetric analysis (TGA Q50, TA instrument).
